# Postoperative Analgesia and Length of Hospital Stay After Surgery for Malignant Pleural Mesothelioma: A Retrospective Observational Study

**DOI:** 10.5812/aapm-150055

**Published:** 2024-12-15

**Authors:** Mayuu Kobata, Kenta Takeda, Mana Taguchi, Hiroai Okutani, Takeshi Ide, Akane Kido, Kouichi Fujimoto, Masaki Hashimoto, Ryusuke Ueki, Munetaka Hirose

**Affiliations:** 1 Department of Anesthesiology, Hyogo Medical University Faculty of Medicine, Nishinomiya, Hyogo, Japan; 2 Department of Thoracic Surgery, Hyogo Medical University Faculty of Medicine, Nishinomiya, Hyogo, Japan

**Keywords:** Hospital Stay, Postoperative Pain, Regional Anesthesia, Surgical Complications

## Abstract

**Background:**

Pleurectomy/decortication (P/D), a surgical procedure for malignant pleural mesothelioma (MPM), is a highly invasive surgery requiring prolonged hospitalization. Previous studies have reported that postoperative analgesia using regional anesthesia contributes to shorter hospital stays after surgery under general anesthesia by reducing acute postoperative pain. However, the association between postoperative analgesia and the length of hospital stay (LOHS) following P/D has not been evaluated.

**Objectives:**

To evaluate the association between postoperative analgesia and postoperative LOHS after P/D.

**Methods:**

This single-institution observational study enrolled consecutive adult patients undergoing P/D under general anesthesia, who postoperatively received either intertransverse process block (ITPB) or continuous intravenous (IV) fentanyl infusion as postoperative analgesia between March 2022 and February 2023.

**Results:**

Among all enrolled patients with ASA physical status II or III (n = 60), postoperative analgesia was administered using either continuous ITPB (n = 19) or continuous IV fentanyl infusion (n = 41). Multivariable logistic regression analysis revealed that postoperative analgesia with continuous ITPB (P = 0.007), a lower incidence of major complications after surgery (P = 0.034), and female sex (P = 0.033) were significantly associated with a shorter postoperative LOHS. In subgroup analysis, patients who received continuous ITPB had significantly lower postoperative LOHS, lower postoperative serum C-reactive protein levels on postoperative day (POD) 3, and reduced acute postoperative pain on POD3 compared to those who received continuous IV fentanyl infusion.

**Conclusions:**

Postoperative analgesia using continuous ITPB appears to be associated with a reduction in LOHS following P/D for MPM under general anesthesia.

## 1. Background

Pleurectomy/decortication (P/D) is a surgical procedure used as part of the multimodal treatment for malignant pleural mesothelioma (MPM) (1, 2). Malignant pleural mesothelioma is a rare and aggressive cancer of the lining around the lung, often associated with asbestos exposure (1-3). The surgery involves removing the pleura, with or without the pericardium and/or diaphragm, and is one of the most invasive procedures among non-cardiac surgeries (3). This invasiveness likely contributes to the long average hospitalization duration (21 days) and the relatively high in-hospital mortality rate (3.2%) (4).

Pleurectomy/decortication for MPM is performed under general anesthesia, with or without regional anesthesia such as epidural block or thoracic paravertebral block (TPVB) (5, 6). Although continuous epidural block for postoperative analgesia has been reported to reduce the rate of major complications after P/D, adverse postoperative events, including orthostatic hypotension, occur in approximately 15% of patients due to the bilateral sympathetic blockade caused by epidural anesthesia (6). Consequently, the development of alternative postoperative analgesia strategies to replace epidural anesthesia is highly desirable for patients undergoing P/D.

Several studies have reported that the addition of regional anesthesia to general anesthesia can shorten the length of hospital stay (LOHS) and reduce acute postoperative pain after cardiothoracic and spinal surgeries (7, 8). The recently developed ultrasound-guided intertransverse process block (ITPB) serves as an alternative to TPVB, offering multilevel blockade for thoracic surgeries. The use of a continuous catheter technique for ITPB has successfully provided postoperative analgesia without serious adverse events (9, 10). Since ITPB performed on the surgical side results in ipsilateral sympathetic blockade only, continuous ITPB is expected to shorten the postoperative hospital stay after P/D while reducing adverse postoperative events.

## 2. Objectives

This retrospective observational study examined patients undergoing P/D for MPM to investigate the potential association between continuous ITPB for postoperative analgesia and postoperative outcomes, including hospital length of stay.

## 3. Methods

### 3.1. Ethics

This observational study was approved by the Ethics Committee of Hyogo Medical University (ethics committee number 3138) on March 4, 2019. The requirement for written informed consent was waived by the institutional ethics committee. This study was conducted in accordance with the principles of the Declaration of Helsinki.

### 3.2. Patients

Hyogo Medical University Hospital is a leading center in Japan with extensive experience in performing P/D surgery for MPM with the goal of achieving a cure. Therefore, this study was conducted exclusively at our institution. Participants included consecutive patients who underwent P/D under general anesthesia between March 2022 and February 2023 at the surgical center of Hyogo Medical University Hospital. The exclusion criteria were: Age < 19 years, American Society of Anesthesiologists-physical status (ASA-PS) ≥ IV, and emergency surgery.

### 3.3. Data Collection

To assess the primary outcome of the association between postoperative analgesia and postoperative LOHS in this cohort study, perioperative data were obtained from our institutional medical records. The collected data included age, sex, Body Mass Index (BMI), emergentness of surgery, ASA-PS, anesthesia management, serum C-reactive protein (CRP) levels measured before surgery and on days 1 and 3 post-surgery, and the durations of intensive care unit (ICU) and hospital stays after surgery.

To evaluate the balance between nociception caused by surgery and anti-nociception from anesthesia, we used Vi-Pros software (Dowell Co. Ltd., Sapporo, Japan) to calculate an average pain response score (mean NR) for each patient throughout their surgery (11). The NR Index is a score ranging from 0 to 1, indicating how the body responds to nociception during surgery under general anesthesia. It is calculated every minute using a formula that takes into account three measurements: Heart rate, systolic blood pressure, and perfusion index. In a previous report, multimodal general anesthesia guided by a nociception monitor, with the NR Index maintained below 0.85 as much as possible, was shown to reduce the development of postoperative complications (12).

### 3.4. Acute Postoperative Pain

To evaluate acute postoperative pain at rest, Numerical Rating Scale (NRS) scores were measured on postoperative days (POD) 1 and 3. The NRS ranged from 0, indicating no pain, to 10, representing the worst imaginable pain.

### 3.5. Postoperative Complications

Postoperative complications were graded according to the Clavien-Dindo classification, which includes five grades from I to V (13). Major complications were defined as Clavien-Dindo grade III or higher, which correspond to complications that require interventions performed under local anesthesia or more invasive measures.

### 3.6. Anesthetic Management During Surgery

Appropriate preoperative fasting was enforced for all patients, and no pre-medications were administered due to the low risk of pulmonary aspiration. Anesthesia was induced with propofol, fentanyl, remifentanil, and rocuronium. A left-sided double-lumen tube was used for intubation. In addition to fentanyl and rocuronium, continuous intravenous (IV) infusions of propofol and remifentanil were used to maintain total IV anesthesia throughout the surgery. The amounts of remifentanil and fentanyl were adjusted to keep the patient’s mean blood pressure within 20% of their baseline blood pressure. Propofol was also used to maintain the Bispectral Index (BIS) between 40 and 60. Hemoglobin concentrations above 10 g·dL⁻¹ were maintained by transfusing red blood cells (RBC) as necessary.

The anesthesiologist in charge determined whether to perform an ITPB or TPVB after induction of anesthesia, based on the patient’s co-morbidities (e.g., coagulopathy, thoracic spine deformity, or cancer invasion at the site of regional anesthesia) or their experience.

In patients who received ITPB, both the transverse process at the Th7 vertebral level and the pleura were identified under ultrasonographic guidance (SONIMAGE HS2; Konica Minolta, Inc., Tokyo, Japan) using a linear probe. An 18-gauge through-the-needle catheter (Contiplex Ultra 360; B BRAUN Co., Germany) was inserted using an in-plane technique, advancing it toward the midpoint between the transverse process and the pleura. The spread of the injectate at the midpoint between the posterior border of the transverse process and the pleura was confirmed by a bolus injection of 20 mL of 0.25% levobupivacaine. The success of the ITPB was subsequently verified through ultrasonographic imaging. Following the single-shot injection, a side-hole catheter (Perifix ONE Catheter; B BRAUN Co., Germany) was inserted 7 - 14 cm into the Th7 - 8 interspace under ultrasound guidance, with the patient in the lateral decubitus position.

Conversely, in patients who received TPVB, an ultrasonography probe was positioned to visualize the spinous and transverse processes of the Th7 vertebra along with the associated costa. With proper visualization, ultrasound-guided TPVB was performed using a single injection of 20 mL of 0.25% levobupivacaine. Depression of the pleura was considered indicative of successful injection of the analgesic agent. The effects of a single injection of ITPB on postoperative analgesia have been reported to be comparable to those of a single injection of TPVB in patients undergoing thoracoscopic surgery (14).

### 3.7. Postoperative Analgesia

All patients were extubated in the operating room and then transferred to the ICU. In patients who received a single injection of ITPB after induction of general anesthesia, no additional local anesthetics were administered via the catheter during surgery. At the end of the surgery, a continuous infusion of 0.125% levobupivacaine at a rate of 4 mL/h was started and continued until POD3 for postoperative analgesia. In contrast, patients who received TPVB did not have a continuous catheter inserted; instead, they received fentanyl through a continuous IV infusion, which began after surgery and continued until POD3. The dose was between 25 and 30 μg/h. For both groups (ITPB and TPVB), if a patient required additional pain relief after surgery, they were given extra medication via IV (acetaminophen), orally (tramadol or loxoprofen), or as a skin patch (fentanyl).

Patients were monitored for postoperative adverse events (e.g., orthostatic hypotension and fluid leakage from the ITPB puncture site), which could potentially lead to the earlier interruption of postoperative analgesia than the planned discontinuation on POD3.

### 3.8. Statistical Analysis

Statistical testing was performed using JMS Pro version 14.2.0 (SAS Institute Inc., Cary, NC, USA) or IBM SPSS Statistics 24 software (IBM Corp., Chicago, IL, USA). A multivariable logistic regression analysis was conducted to assess the association between ITPB and LOHS after surgery. To exclude confounding effects between ITPB and perioperative variables, we selected preoperative and perioperative variables as candidate variables for the analysis. If multicollinearity was detected between these variables [based on a variance inflation factor (VIF) of less than 10], the variables were not included in the analysis (15). The results are presented as odds ratios with 95% confidence intervals (CIs).

### 3.9. Subgroup Analysis

We divided the patients into two groups: Those who received postoperative analgesia with continuous ITPB and those who received continuous IV fentanyl infusion. Comparisons between the two groups were performed using the unpaired *t*-test, Wilcoxon rank sum test, or chi-squared test, as appropriate. Normality of the data was assessed using normal quantile plots, and values of P < 0.05 were considered statistically significant.

## 4. Results

### 4.1. Patients’ Characteristics and Perioperative Variables

This study adhered to the STROBE guidelines. Sixty patients undergoing P/D for MPM under general anesthesia were enrolled, with either a single-shot ITPB followed by postoperative continuous ITPB or a single-shot TPVB followed by postoperative continuous IV fentanyl infusion, between March 2022 and February 2023. Table 1 shows the perioperative data. The mean LOHS was 22 [18 - 26] days after P/D.

**Table 1.** Perioperative Variables

Preoperative Variables	Values ^a^
**Age, y**	69 ± 8
**Sex**	
Male	52 (86.7)
Female	8 (13.3)
**BMI, kg.m** ^**2**^	24.1 ± 3.2
**ASA-PS**	
II	10 (16.7)
III	50 (83.3)
**Preoperative CRP level, mg.dL** ^**-1**^	0.22 ± 0.30
**Single-shot regional block after induction of anesthesia**	
ITPB	19 (31.7)
TPVB	41 (68.3)
**Intraoperative**	
Continuous remifentanil dose, μg.kg^-1^.min^-1^	0.154 ± 0.042
Total dose of fentanyl, μg.kg^-1^	17.7 ± 5.6
Total dose of rocuronium, μg.kg^-1^	3.1 ± 0.8
Length of surgery, min	380 ± 89
Length of anesthesia, min	488 ± 92
Volume of blood loss, mL	1783 ± 1302
Volume of RBC transfusion, mL	901 ± 650
Urine volume, mL	644 ± 492
**Postoperative**	
Postoperative analgesia	
Continuous ITPB	19 (31.7)
Continuous IV fentanyl infusion	41 (68.3)
Length of postoperative ICU stay, days	3 [2 - 4]
Length of postoperative hospital stay, days	22 [18 - 26]
NRS at rest	
POD1	1 [0 - 2]
POD3	1 [0 - 3]
CRP level, mg.dL^-1^	
POD1	5.38 (1.98)
POD3	7.90 (6.12)
Clavien-Dindo grade	
< III	15 (25.0)
≥ III	45 (75.0)

Abbreviations: ASA-PS, American Society of Anesthesiologists-Physical Status; BIS, Bispectral Index; BMI, Body Mass Index; CRP, C-reactive protein; ICU, intensive care unit; ITPB, intertransverse process block; IV, intravenous; MTP, midpoint of the transverse process to pleura; NRS, Numerical Rating Scale; POD, postoperative day; RBC, red blood cells; SD, standard deviation; TPVB, thoracic paravertebral block.

^a^ Values are expressed as No. (%), means ± SD, or median [25th - 75th percentile].

Nineteen patients (31.7%) received continuous ITPB after surgery, and 41 patients (68.3%) received continuous IV fentanyl infusion. There were no postoperative adverse events that led to the early interruption of either continuous ITPB or continuous IV fentanyl infusion. The incidence of postoperative major complications was 75.0%. Major complications occurred in 45 patients, including air leak (n = 42), reoperation (n = 1), brain infarction (n = 1), and renal failure (n = 1).

### 4.2. Association Between Postoperative Analgesia and Length of Hospital Stay After Surgery

Since the mean postoperative stay after P/D was 21 days (4), we employed multivariable logistic regression analyses to assess the relationship between postoperative analgesia and LOHS ≥ 21 days after surgery. Eight preoperative and intraoperative variables were selected as candidate variables: Age ≥ 70 years, male sex, BMI ≥ 25 kg/m², ASA-PS ≥ III, preoperative CRP levels ≥ 1.00 mg/dL, continuous ITPB, mean NR ≥ 0.83, and RBC transfusion ≥ 1 200 mL (6). Additionally, Clavien-Dindo grade ≥ III was also selected as a postoperative candidate variable. No multicollinearity between candidate variables was observed. The analysis revealed that the absence of continuous ITPB, Clavien-Dindo grade ≥ III, and male sex were independent risk factors for a postoperative LOHS ≥ 21 days (Table 2). 

**Table 2.** Perioperative Variables Related to Postoperative Length of Hospital Stay ≥ 21 Days

Pre- and Intraoperative Variables	Odds Ratio [95% CI]	P-Value
**Age, y**		0.951
< 75	Ref	
≥ 75	1.05 [0.23 - 4.86]	
**Sex (male) **	8.83 [1.19 - 65.3]	0.033 ^a^
**BMI, kg.m** ^**2**^		0.105
< 25	Ref	
≥ 25	0.32 [0.08 - 1.27]	
**ASA-PS**		0.730
< III	Ref	
≥ III	1.34 [0.25 - 7.21]	
**Preoperative CRP level, mg.dL** ^**-1**^		0.517
< 1.00	Ref	
≥ 1.00	0.37 [0.02 - 7.52]	
**Continuous ITPB**	0.14 [0.03 - 0.59]	0.007 ^a^
**Mean NR**		0.802
< 0.83	Ref	
≥ 0.83	1.23 [0.24 - 6.46]	
**RBC transfusion volume, mL**		0.212
< 1200	Ref	
≥ 1200	0.31 [0.05 - 1.97]	
**Clavien-Dindo grade**		0.034 ^a^
< III	Ref	
≥ III	5.84 [1.14 - 29.97]	

Abbreviations: BMI, Body Mass Index; ITPB, intertransverse process block; CRP, C-reactive protein; RBC, red blood cells; ASA-PS, American Society of Anesthesiologists-Physical Status.

^a^ Significant at P < 0.05.

For the subgroup analysis, we divided the patients into two groups: The continuous ITPB group (n = 19) and the continuous IV fentanyl infusion group (n = 41) (Table 3). There were no significant differences in preoperative variables between the two groups. Among intraoperative variables, the mean NR index during surgery was significantly higher in patients who received continuous ITPB compared to those who received continuous IV fentanyl infusion. Conversely, the continuous remifentanil dose was significantly lower in patients with continuous ITPB than in those with continuous IV fentanyl infusion. There were no significant differences in the length of surgery, blood loss, or transfusion volume between the two groups.

**Table 3.** Comparisons of Perioperative Variables Between Patients with Continuous Intravenous Fentanyl Versus Continuous Intertransverse Process Block ^a,^ ^b^

Perioperative Variables	Continuous IV Fentanyl Infusion (n = 41)	Continuous ITPB (n = 19)	P-Value
**Preoperative**			
Age, y	70 (7)	68 (9)	0.319
Sex			0.703
Male	36 (87.8)	16 (84.2)	
Female	5 (12.2)	3 (15.8)	
BMI, kg.m^2^	24.0 (3.2)	24.4 (3.1)	0.704
ASA-PS			0.172
II	5 (12.2)	5 (26.3)	
III	36 (87.8)	14 (73.7)	
CRP level, mg.dL^-1^	0.24 (0.34)	0.17 (0.20)	0.386
**Intraoperative**			
Mean NR	0.849 (0.023)	0.868 (0.031)	0.009 ^c^
Continuous remifentanil dose, μg.kg^-1^.min^-1^	0.163 (0.040)	0.134 (0.041)	0.012 ^d^
Total dose of fentanyl, μg.kg^-1^	17.0 (6.1)	19.1 (4.3)	0.189
Total dose of rocuronium, μg.kg^-1^	3.1 (0.9)	3.1 (0.8)	0.848
Length of surgery, min	395 (84)	347 (93)	0.054
Length of anesthesia, min	500 (85)	463 (103)	0.149
Blood loss, mL	1760 (864)	1832 (1974)	0.845
RBC transfusion volume, mL	892 (367)	922 (1055)	0.873
Urine volume, mL	643 (455)	647 (577)	0.975
**Postoperative**			
Length of postoperative ICU stay (days)	3 [2 – 4]	4 [3 – 4]	0.032 ^d^
Length of postoperative hospital stay (days)	22 [20 – 31]	17 [14 – 24]	0.005 ^c^
NRS score for pain at rest			
POD1			0.328
< 4	39 (95.1)	19 (100.0)	
≥ 4	2 (4.9)	0 (0.0)	
POD3			0.045 ^d^
< 4	36 (87.8)	19 (100.0)	
≥ 4	5 (12.2)	0 (0.0)	
CRP level, mg.dL^-1^			
POD1	5.38 (2.21)	5.39 (1.40)	0.995
POD3	9.14 (6.50)	5.07 (4.04)	0.017 ^d^
Clavien-Dindo grade			0.627
< III	11 (26.8)	4 (21.1)	
≥ III	30 (73.2)	15 (78.9)	

Abbreviations: IV, intravenous; ITPB, intertransverse process block; NRS, Numerical Rating Scale; CRP, C-reactive protein; POD, postoperative day; RBC, red blood cells; ASA-PS, American Society of Anesthesiologists-Physical Status; ICU, intensive care unit.

^a^ Values are expressed as No. (%), means ± SD, or median [25th - 75th percentile].

^b^ Comparisons of two variables were performed using the unpaired *t*-test, Wilcoxon rank-sum test, or chi-square test.

^c^ Significant at P < 0.01.

^d^ Significant at P < 0.05.

Regarding postoperative variables, the LOHS after surgery was significantly shorter in patients with continuous ITPB compared to those with continuous IV fentanyl infusion. Although the duration of ICU stay after surgery was significantly longer in patients with continuous ITPB than in those with continuous IV fentanyl infusion, both the NRS values at rest on POD3 and serum CRP levels on POD3 were significantly lower in patients with continuous ITPB compared to those with continuous IV fentanyl infusion.

## 5. Discussion

Several previous studies have reported that risk factors for prolonged hospital stay after non-cardiac surgery include postoperative complications, male sex, prolonged duration of surgery, older age, and metastasis (16-18). In patients with MPM undergoing P/D, both a higher incidence of major complications after surgery and male sex were associated with a longer hospital stay in the present study. Furthermore, the absence of continuous ITPB for postoperative analgesia was associated with prolonged hospital stay in this study. A previous meta-analysis showed that postoperative analgesia using regional anesthesia in patients undergoing cardiothoracic surgery under general anesthesia decreased LOHS by reducing acute postoperative pain (7). Another meta-analysis reported that erector spinae plane block decreased postoperative hospital stay and reduced postoperative pain, opioid consumption, and nausea and vomiting after lumbar spine surgery (8). Although the precise mechanisms underlying the beneficial effects of regional anesthesia on postoperative LOHS have not been fully explained, the latter study suggested that lower opioid consumption after surgery might contribute to this effect (8).

In the subgroup analysis, we compared perioperative variables between patients who received continuous ITPB and those who received continuous IV fentanyl infusion. We found that continuous doses of remifentanil during surgery, serum CRP levels on POD3, acute postoperative pain on POD3, and the LOHS after surgery were significantly lower in patients with continuous ITPB than in those with continuous IV fentanyl infusion. In contrast, the mean NR Index during surgery was significantly higher in patients with continuous ITPB than in those with continuous IV fentanyl infusion, and the length of ICU stay after surgery was significantly longer in patients with continuous ITPB than in those with continuous IV fentanyl infusion. There were no significant differences in the incidence of major complications within 30 days after surgery between the two patient groups (Table 3). Previous studies have shown that a higher postoperative CRP level, which indicates greater surgical invasiveness (19), is a valuable predictor of major complications after non-cardiac surgery (20). However, no such associations were observed in this study. A subgroup analysis may provide useful information, and can also lead to misleading results (21). Although a reduction in postoperative pain in patients with continuous ITPB might partly contribute to the decrease in postoperative LOHS, this subgroup analysis did not identify any mechanisms that clearly explain the association between postoperative analgesia and LOHS after P/D.

A limitation of this study is the small number of patients. Although Hyogo Medical University Hospital is a major center in Japan known for its expertise and high volume of P/D surgeries (4), the number of these surgeries performed each year is limited, and the number of patients who received continuous ITPB for postoperative analgesia is still small, as ITPB was only recently introduced (9). Another limitation of this study is that the method of regional anesthesia performed before skin incision was a single injection of ITPB in patients who received continuous ITPB postoperatively, which differed from the single injection of TPVB used in patients with continuous postoperative IV fentanyl infusion. Although the effects of ITPB and TPVB on postoperative pain are reportedly comparable in patients with the same method of postoperative analgesia (14), the mean NR Index values, which represent the balance between nociception caused by surgical invasiveness and anti-nociception from anesthesia during surgery, significantly differed between these two groups. Thus, further investigation, including a larger number of patients with the same intraoperative anesthetic management, is needed to better understand the mechanisms behind the association between postoperative analgesia and hospitalization in future studies.

### 5.1. Conclusions

Postoperative analgesia using continuous ITPB is likely associated with a decrease in LOHS and a reduction in acute postoperative pain after P/D for MPM, compared to continuous IV fentanyl infusion.

## Data Availability

Data will be made available upon submission of a reasonable written request to the corresponding author.
